# c-Jun N-Terminal Phosphorylation: Biomarker for Cellular Stress Rather than Cell Death in the Injured Cochlea^1^,^2^,^3^

**DOI:** 10.1523/ENEURO.0047-16.2016

**Published:** 2016-05-24

**Authors:** Tommi Anttonen, Anni Herranen, Jussi Virkkala, Anna Kirjavainen, Pinja Elomaa, Maarja Laos, Xingqun Liang, Jukka Ylikoski, Axel Behrens, Ulla Pirvola

**Affiliations:** 1 Department of Biosciences, University of Helsinki, 00014 Helsinki, Finland; 2 Finnish Institute of Occupational Health, 00251 Helsinki, Finland; 3 Helsinki Ear Institute, 00420 Helsinki, Finland; 4 The Francis Crick Institute and King's College London, London SE1 1UL, UK

**Keywords:** c-Jun phosphorylation, cell death, hair cell, inner ear, noise, supporting cell

## Abstract

Prevention of auditory hair cell death offers therapeutic potential to rescue hearing. Pharmacological blockade of JNK/c-Jun signaling attenuates injury-induced hair cell loss, but with unsolved mechanisms. We have characterized the c-Jun stress response in the mouse cochlea challenged with acoustic overstimulation and ototoxins, by studying the dynamics of c-Jun N-terminal phosphorylation. It occurred acutely in glial-like supporting cells, inner hair cells, and the cells of the cochlear ion trafficking route, and was rapidly downregulated after exposures. Notably, death-prone outer hair cells lacked c-Jun phosphorylation. As phosphorylation was triggered also by nontraumatic noise levels and none of the cells showing this activation were lost, c-Jun phosphorylation is a biomarker for cochlear stress rather than an indicator of a death-prone fate of hair cells. Preconditioning with a mild noise exposure before a stronger traumatizing noise exposure attenuated the cochlear c-Jun stress response, suggesting that the known protective effect of sound preconditioning on hearing is linked to suppression of c-Jun activation. Finally, mice with mutations in the c-Jun N-terminal phosphoacceptor sites showed partial, but significant, hair cell protection. These data identify the c-Jun stress response as a paracrine mechanism that mediates outer hair cell death.

## Abstract (precis)

Prevention of auditory hair cell death offers therapeutic potential to rescue hearing. Pharmacological blockade of JNK/c-Jun signaling attenuates injury-induced hair cell loss, but with unsolved mechanisms.

## Visual Abstract

**Figure:**
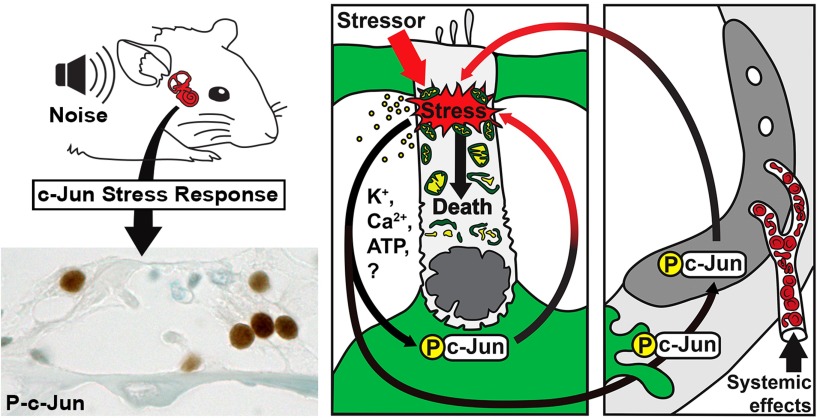

## Significance Statement

The current study reveals a detailed time course and cellular pattern of the c-Jun stress response in the cochlea following traumas. It identifies N-terminal phosphorylation of c-Jun as a biomarker for acute cellular stress. The results that were obtained suggest a novel function for c-Jun phosphorylation in the adult cochlea, not as an intrinsic mediator of cell death, but as a part of a paracrine response that regulates hair cell death following traumas. These results are important for the development of protective therapies against hair cell loss.

## Introduction

Cells undergo a wide range of molecular changes in response to stress. Stress signaling pathways enable cells to make adaptive responses to maintain tissue homeostasis and to promote cell survival. Upon intolerable levels of stressors, activated stress signaling fuels into proapoptotic cascades, either cell-autonomously or via paracrine mechanisms (Fulda et al., 2010). The stress-activated c-Jun N-terminal protein kinase (JNK)/c-Jun pathway mediates cell death and inflammation in a context-dependent fashion. In addition to pathophysiological processes, JNK/c-Jun activation regulates developmental events, particularly programmed cell death. It is a sequential kinase-signaling pathway where mixed lineage kinases (MLKs) activate JNKs by phosphorylation. Activated JNKs in turn phosphorylate their cytoplasmic and nuclear targets. A predominant nuclear JNK substrate is c-Jun, a transcription factor of the activator protein 1 complex (Raivich, 2008; Coffey, 2014). Phosphorylation of c-Jun N-terminal serines 63 and 73 by JNKs is linked to traumas and apoptotic death, and, therefore, antibodies detecting this phosphorylation have been widely used as an indicator of stress-induced JNK/c-Jun signaling (Yang et al., 1997; Herdegen et al., 1998; Behrens et al., 1999; Besirli et al., 2005; Brecht et al., 2005).

Acoustic overstimulation and ototoxic drugs cause hearing loss. Primary targets of these stressors are the mechanosensory hair cells of the auditory sensory epithelium, the organ of Corti of the cochlea. Of the two types of hair cells, outer hair cells (OHCs) are most sensitive to stressors, while inner hair cells (IHCs) are typically killed only by severe insults. The cochlear stress response is manifold. Stressors kill hair cells not only by direct actions. They can also impair cochlear ion homeostasis and in this way indirectly cause hair cell death. Local homeostasis in the organ of Corti is maintained by glial-like supporting cells. These cells scavenge potassium ions that upon sound stimulation enter the sensory epithelium via hair cells from the endolymph. Potassium is then transported back to the endolymph through a recycling route extending from the nonsensory cells of the organ of Corti to the cochlear lateral wall (Kikuchi et al., 2000). Stressors can impair potassium trafficking at different sites along this path (Wang et al., 2002; Wangemann, 2006; Yamaguchi et al., 2014). Supporting cells have also other important supportive functions. Supporting cells around IHCs take up the neurotransmitter glutamate to prevent excitotoxic damage to afferent nerve endings. Additionally, upon hair cell loss, supporting cells are critical in healing the sensory epithelial surface, thereby preventing detrimental leakage of the endolymph into the epithelium (Monzack and Cunningham, 2013; Anttonen et al., 2014).

In the injured cochlea, the potential of modulating different stress signaling pathways to prevent hair cell death has been widely studied. JNK/c-Jun signaling is one of the therapeutically promising targets, based on the findings that pharmacological blockade of MLK or JNK activation confers protection against hair cell death (Pirvola et al., 2000; Ylikoski et al., 2002; Wang et al., 2003). Still, the role and basic mechanism of JNK/c-Jun signaling in the cochlea remain unsolved. Herein, we have examined the pattern and function of this signaling in the noise- and ototoxin-exposed adult cochlea and in the developing cochlea. With expression data and genetic evidence, we present novel insights into the mechanisms of JNK/c-Jun signaling. The results underscore the importance of understanding the pleiotropic mechanisms of this signaling if its pharmacological modulation for therapeutic purposes is realized.

## Materials and Methods

### Mice

Seven-to-eight-week old CBA/Ca mice (Harlan Laboratories) of both sexes were used to study c-Jun expression and N-terminal phosphorylation in nonexposed cochleas and in cochleas exposed to noise and ototoxic drugs, including acoustic preconditioning experiments. CBA/Ca mice at postnatal day 0 (P0), P6, and P12 were used for immunostaining of the immature cochlea. *Gfi1^GFP/GFP^* knock-in mice and control littermates were analyzed at P0 and at 4 weeks of age. Generation and genotyping of these mutant mice have been described previously (Yücel et al., 2004). *JunAA/AA* mutant mice and control littermates had a mixed genetic background (C57BL/6,129/S6, CBA/Ca; Behrens et al., 1999). All mice were bred from in-house breeding pairs. All animal work was conducted according to relevant national and international guidelines. Approval for animal experiments was obtained from the National Animal Experiment Board. Cochleas from at least three mice per age and per genotype were used for immunohistochemistry. In addition, cochleas from three individuals of both the *Gfi1^GFP/GFP^* and control *Gfi1^GFP/+^* genotypes at P0 were prepared for whole-mount specimens.

### Ototoxic trauma

Hair cell loss was induced by a single subcutaneous injection of 1 mg/g kanamycin (Sigma-Aldrich) followed by a single intraperitoneal injection of 0.4 mg/g furosemide (Fresenius Kabi) according to an established protocol (Oesterle et al., 2008; Taylor et al., 2008). The interval between the injections was 30 min. Animals were killed at 2, 8, 20, and 60 h after the ototoxic challenge. Cochleas from a minimum of three mice per postexposure time point were processed for immunohistochemistry.

### Noise exposures and sound preconditioning

Mice were exposed for 1 or 4 h or 15 min to octave-band noise centered at 8-16 kHz at 85, 91, or 106 dB SPL. Cochleas were evaluated immediately, and at 6 h, 20 h, and 7 d after exposure. Sound preconditioning was performed for 1 h at 91 dB SPL, and, after an interval of 12 h, mice were exposed for 1 or 4 h to 106 dB SPL and were evaluated immediately thereafter. Exposures were performed in a ventilated, self-built sound chamber (40 × 44 × 82 cm). Sound was produced with two active speakers (8130A Digital Bi-Amplifier Monitoring System, Genelec) mounted side by side 2 cm above a laboratory animal cage for rodents. Speakers were connected with NuForce icon µDAC2 to a laptop playing the sound continuously. The cage was subdivided into four smaller cages (9 × 16 × 9 cm) for each individual. In these cages, restraint stress was avoided, as mice were able to turn and move. A minimum of three mice per SPL, per noise exposure duration, and per postexposure time point were used to perform immunohistochemistry on cochlear sections. Eight noise-exposed mice were used for immunocytochemistry on whole-mount specimens. Four adult individuals of both *Gfi1^GFP/GFP^* and *Gfi1^GFP/+^* knock-in mice were exposed to noise, and their cochleas were prepared for immunohistochemistry. Eight mice were used for preconditioning experiments, half of them for the shorter and the other half for the longer traumatizing noise exposure. In most of these cases, both cochleas of each animal were histologically analyzed. Concerning *JunAA/AA* and control littermates, eight mice of both genotypes were exposed for 6 h to 110 and 115 dB SPL each, and were analyzed 16 d postexposure. One cochlea per animal was processed for resin-embedded specimens.

### Immunohistochemistry and ApopTag staining

Cochleas were perilymphatically fixed with 4% paraformaldehyde (PFA) in PBS and immersed in the fixative overnight at +4°C. P6, P12, and adult cochleas were decalcified in 0.5 m EDTA, pH 7.5. Cochleas were embedded into paraffin (Historesin IM, Thermo Scientific). Five-micrometer-thick sections were cut in the midmodiolar plane through cochleas. After deparaffinization, epitopes were unmasked by microwave heating (900 W) in 10 mm citrate buffer, pH 6.0, for 10 min of boiling. Sections were blocked for 30 min with 10% goat serum (Jackson ImmunoResearch) in PBS containing 0.25% Triton X-100 (PBS-T). Incubation with primary antibodies diluted in PBS-T was performed for 48 h at +4°C. The following primary antibodies were used: c-Jun, phospho-c-Jun Serine 73, phospho-c-Jun Serine 63, cleaved caspase-3 (all rabbit monoclonals; Cell Signaling Technology); and myosin 7a (rabbit polyclonal; Proteus Biosciences). Detection was performed with Vectastain Elite ABC Kit and diaminobenzidine substrate (DAB Detection kit; Vector Laboratories). ApopTag Peroxidase In Situ Apoptosis Detection Kit (Millipore) was used to detect DNA single- and double-stranded breaks associated with late stages of apoptosis. Sections were counterstained with 3% methyl green and mounted in Permount (Fisher Scientific). A part of consecutive sections was stained with hematoxylin (Shandon Instant Hematoxylin, Thermo Scientific).

### Whole-mount specimens

Cochleas fixed with PFA and decalcified with EDTA were cut in the midmodiolar plane in half, such that the coiled organ of Corti was separated into four pieces. These pieces were dissected clean from the surrounding tissue, and the tectorial membrane was removed. For immunofluorescence, whole mounts were blocked for 30 min with 10% donkey serum in PBS-T, followed by a 48-h-long incubation at +4°C with appropriate primary antibodies in PBS-T. The following primary antibodies were used: Sox2 (goat polyclonal; Santa Cruz Biotechnology); and phospho-c-Jun Serine 73. Secondary antibodies conjugated to Alexa Fluor 568, 594, or 647 were used for detection. After antibody incubations, F-actin filaments were visualized using Oregon Green 514-conjugated phalloidin (1:400 in PBS-T). Nuclei were stained with DAPI. ProLong Gold Antifade Reagent was used for mounting (all from ThermoFisher Scientific).

### Light microscopy

Sections were analyzed with bright-field and differential interference contrast (DIC) optics under BX61 Microscope equipped with UPlanApo 10×, 20×, and 60× objectives. Images were acquired through DP73 CCD Color Camera and CellSens Software (all from Olympus). Confocal images were acquired using a Leica TCS SP5 laser**-**scanning microscope with Plan Apochromat 10×/0.40 numerical aperture (NA) and 63×/1.3 NA glycerol objectives. The acquisition software was Leica LAS AF. ImageJ was used for the creation of *z*-projections.

### Cochlear frequency mapping in whole mounts

Cochlear frequency mapping in whole-mount specimens was performed with the Measure_Line.class ImageJ plugin (available with a manual from Eaton–Peabody Laboratories Histology Resources) that is based on calculations performed by Müller et al. (2005).

### Graphical reconstruction of the cochlea from paraffin sections and cytocochleograms

A graphical model map for the quantification of c-Jun S73 and c-Jun immunoreactivities and hair cell loss was built from a nontreated adult cochlea that was cut into 5-μm-thick paraffin sections in the midmodiolar plane and stained with hematoxylin. Every eighth consecutive section was imaged. Section images were stacked, arranged, and spatially adjusted such that 3D segmentation was possible. The organ of Corti was manually segmented from the image stack and visualized with Amira (FEI). Percentual distances from the base of the organ of Corti were measured to every originally sectioned plane of the organ of Corti, and corresponding frequency values were mapped with the Measure_Line ImageJ plugin (see above). With these data, a 2D map of the coiled organ of Corti was drawn where each sectioned area of this organ was displayed with the corresponding frequency value together with a grid, each square representing a certain hair cell or supporting cell type. After completing each map, the gained data were redrawn into a linear cytocochleogram for visualization purposes. In the cytocochleograms, light and dark blue squares represent the weak and intense immunoreactivities, respectively, shown in examples of c-Jun S73-stained nuclei (see Fig. 7). Black squares represent lost hair cells. One representative cochlea of each condition is shown as a cytocochleogram.

### Resin-embedded cytocochleograms

Sixteen days after noise exposure (6 h at 110 or 115 dB SPL), the cochleas of *JunAA/AA* and *Junwt/wt* mice were embedded in epoxy resin and processed for surface preparations. These specimens were analyzed under Olympus BX61 Microscope equipped with DIC optics. Hair cells were characterized as missing if no cuticular plate and stereocilia in the normal apical location were observed (replaced by supporting cells scars) and if the respective hair cell nucleus was absent. A 60× objective was used for cell counting. The number of missing hair cells per cochlea were counted in nonexposed (where the number of OHCs averaged 2300) and in noise-exposed mutant and wild-type mice.

### Image analysis and editing

Image analysis and *z*-projection images were performed with the public domain image**-**processing program ImageJ (National Institutes of Health) equipped with Bio-Formats Importer plugin (Open Microscopy Environment). Image editing was performed with Adobe Photoshop CS6.

### Statistical analysis

The normality of the *JunAA* dataset was tested with the Saphiro–Wilk normality test. *p* Values were obtained with a two-tailed two-sample *t* test, and *p* values <0.05 were considered to be significant. Statistical analysis was conducted with Origin Pro 8.6.

## Results

### c-Jun is phosphorylated in the developing cochlea

**Figure 1.**
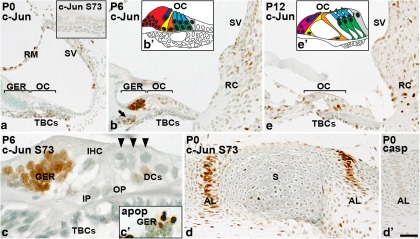
c-Jun expression and S73 phosphorylation during development. ***a***, At P0, tympanic border cells and scattered cells of the lateral wall and Reissner’s membrane express c-Jun. Inset reveals that this expression is not linked with c-Jun S73 phosphorylation, shown in the tympanic border cell region. ***b***, At P6, c-Jun is expressed in the greater epithelial ridge and in Schwann cells of the cochlear nerve (arrow). ***b'***, Schematic representation of the organ of Corti at P6: greater epithelial ridge (red), inner hair cell (blue), outer hair cells (cyan), inner pillar cell (yellow), outer pillar cell (orange), Deiters’ cells (green), and Hensen cell (gray). ***c***, c-Jun S73 is phosphorylated in the greater epithelial ridge at P6. Arrowheads mark outer hair cells. ***c'***, An adjacent, ApopTag-stained section shows apoptotic fragments in the same area. ***d***, At P0, the annular ligament of stapes shows c-Jun S73 phosphorylation. ***d'***, Apoptotic cells are not present in this structure, as evidenced by cleaved caspase-3 staining. ***e***, At P12, c-Jun is expressed in Deiters’ cells of the organ of Corti and in the root cell area of the lateral wall. ***e'***, Schematic representation of the cytoarchitecture of the mature organ of Corti: inner border cells (magenta). Other color codes as in ***b'***. AL, Annular ligament of stapes; apop, ApopTag-staining; casp, cleaved caspase-3; DC, Deiters’ cell; IP, inner pillar cell; OC, organ of Corti; OP, outer pillar cell; TBC, tympanic border cell; RC, root cell; RM, Reissner’s membrane; S, stapes; SV, stria vascularis; GER, greater epithelial ridge. Scale bar: (in ***d'***) ***a***, ***a'***, ***b***, ***d***, ***d'***, ***e***, 40 µm; (in ***d'***) ***c***, ***c'***, 10 µm.

We first studied whether c-Jun is expressed and phosphorylated in the cochlea undergoing postnatal development (Fig. 1). Paraffin sections were stained with antibodies against total c-Jun and against its N-terminal serine 73 phosphorylated form (c-Jun S73). A few studies have shown that the c-Jun S73 antibody recognizes other nuclear JNK substrates besides c-Jun (Besirli et al., 2005; Brecht et al., 2005). We found that cochlear cells with c-Jun S73 phosphorylation invariably expressed c-Jun. Also, antibodies against c-Jun S73 and c-Jun S63 (data not shown) produced comparable staining patterns. Thus, these results suggest that the c-Jun S73 immunoreactivity obtained in our experiments is specific to c-Jun.

In the cochlea at P0 and P6, Schwann cells of the cochlear nerve, epithelial cells of the Reissner’s membrane and the mesenchymal tympanic border cells beneath the organ of Corti showed c-Jun expression, but not c-Jun S73 phosphorylation. Cells of the organ of Corti were negative for both signals (Fig. 1*a–c*). The greater epithelial ridge (GER), located medially to the organ of Corti, regresses through apoptosis at the end of the first postnatal week. At P6, the abrupt induction of both c-Jun and its S73 phosphorylation were seen in the GER, together with apoptotic debris (Fig. 1*b–c'*). These findings in the GER are consistent with those of prior studies showing the involvement of phosphorylated c-Jun in apoptosis of developing tissues (Sun et al., 2005). In the P0 inner ear, in addition to the cochlea, strong c-Jun and c-Jun S73 immunoreactivities were localized to the developing annular ligament of stapes, located at the future oval window at the base of the cochlea. These mesenchymal cells did not show signs of apoptosis, demonstrating that c-Jun N-terminal phosphorylation can have functions other than cell death regulation, consistent with the data on bone and joint development during embryogenesis (Fig. 1*d*,*d'*; Kan and Tabin, 2013).

### c-Jun is expressed in the juvenile and adult organ of Corti and lateral wall

To find out whether c-Jun is expressed and phosphorylated at the time of onset of hearing in mice, analysis was performed at P12. Compared with P6, c-Jun was upregulated in two types of supporting cells of the organ of Corti, the Deiters’ and pillar cells. In the lateral wall, scattered epithelial and mesenchymal cells expressed c-Jun (Fig. 1*e*,*e'*). This expression pattern of basal c-Jun was maintained at adulthood (see Fig. 3*a*). Supporting cells of the lower half of the cochlea showed more prominent c-Jun expression compared with those of the upper half of the cochlea (data not shown). c-Jun S73 staining was found both at P12 and in adulthood only in a small number of cells located in a scattered manner within the supporting cell population of the organ of Corti and in the lateral wall (see Fig. 3*b*). Thus, c-Jun is not significantly phosphorylated under normal physiological conditions in these structures.

### c-Jun is phosphorylated in developing hair cells upon disturbed differentiation

**Figure 2.**
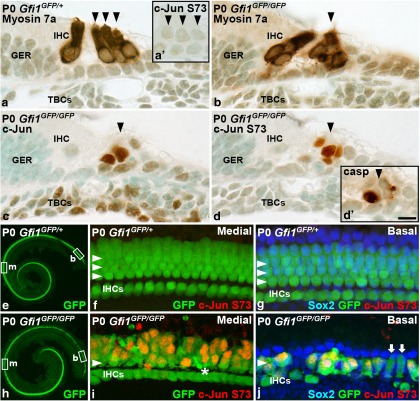
The c-Jun stress response in *Gfi1*-depleted developing hair cells. ***a***, Cochlear hair cells of heterozygous *Gfi1^GFP/+^* mice at P0 have normal morphology, revealed by myosin 7a immunostaining. Arrowheads mark outer hair cells. ***a'***, c-Jun S73 immunoreactivity is not detected in the organ of Corti of these control animals. ***b***, In homozygous *Gfi1^GFP/GFP^* mice, hair cell morphology is severely distorted. ***c***, ***d***, c-Jun is expressed and phosphorylated in the organ of Corti of these mutants. Note that tympanic border cells show basal c-Jun expression, but its S73 phosphorylation is absent. ***d'***, Cleaved caspase-3-positive apoptotic fragments in the hair cell region, shown in an adjacent section. ***e***, In cochleas of *Gfi1^GFP/+^* mice, the *GFP* reporter marks hair cells. Boxed areas indicate the location of images in ***f*** and ***g***. ***f***, In the medial turn of these control specimens, GFP-positive hair cells (arrowheads) lack c-Jun S73 phosphorylation. ***g***, Also in the basal turn, where cellular differentiation is more advanced, GFP-positive hair cells lack the S73 signal. It is also absent from Sox2-positive supporting cells. ***h***, In cochleas of *Gfi1^GFP/GFP^* mice, the medial turn shows scattered hair cell loss, while this loss is more severe in the basal turn, consistent with the basal-to-medial-turn progression of the death of *Gfi1*-depleted hair cells. Boxed areas indicate the location of images in ***i*** and ***j***. ***i***, In the medial turn, the morphology of GFP-positive outer hair cells is distorted, and c-Jun is phosphorylated in these degenerating cells. GFP-positive apoptotic fragments (asterisk) can be seen. ***j***, Only a few GFP-positive hair cells are left in the basal turn. Note that the GFP-positive hair cells lacking c-Jun S73 phosphorylation (arrows) are morphologically quite intact. Sox2-expressing supporting cells lack c-Jun S73 immunoreactivity. b, Basal turn; casp, cleaved caspase-3; m, medial turn; TBC, tympanic border cell; IHCs, inner hair cells; GER, greater epithelial ridge. Scale bar: (in ***d'***) ***a–d'***, 10 µm; (in ***d'***) ***e***, ***h***, 385 µm; (in ***d'***) ***f***, ***g***, ***i***, ***j***, 15 µm.

During cochlear development, hair cells die if their differentiation program is disturbed. To find out whether c-Jun signaling regulates this apoptotic death, mice depleted for *Gfi1* (*growth factor independent 1*) were analyzed. The transcription factor Gfi1 is expressed in the developing cochlear hair cells and is critical for their differentiation, as evidenced in *Gfi1*-inactivated mice by massive hair cell apoptosis around birth, shortly after the onset of the differentiation of these cells (Wallis et al., 2003; Kirjavainen et al., 2008). At P0, the organ of Corti of the *Gfi1^GFP/GFP^* knock-in mice (Yücel et al., 2004) showed disorganized rows of hair cells, marked by myosin 7a, compared with wild-type and *Gfi1^GFP/+^* mice (Fig. 2*a*,*b*). This cellular disorganization was accompanied by c-Jun expression and S73 phosphorylation, as opposed to control littermates (Fig. 2*a'*,*c*,*d*). Also, colabeling experiments in whole-mount surface specimens demonstrated the absence of c-Jun and c-Jun S73 signals in the organ of Corti of control mice (Fig. 2*e–g*). In the *Gfi1^GFP/GFP^* mutant mice, these signals were localized to GFP-positive hair cells, while Sox2-positive supporting cells were negative (Fig. 2*h–j*; data not shown). Thus, c-Jun signaling is not involved in hair cell differentiation around birth. Rather, the induction of c-Jun expression and phosphorylation appears to regulate apoptosis of hair cells stressed by cell-intrinsic genetic perturbations (Fig. 2*c*–*d'*).

### Acoustic overstimulation triggers c-Jun phosphorylation in the organ of Corti and lateral wall

**Figure 3.**
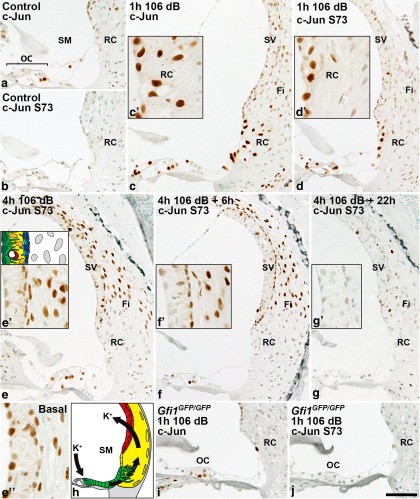
Acoustic overstimulation triggers an acute and transient c-Jun stress response in the potassium-recycling route of the adult cochlea. ***a***, Scattered pillar and Deiters’ cells of the adult organ of Corti express c-Jun, together with a few cells in the lateral wall. ***b***, c-Jun is only sporadically phosphorylated in cells of the nontraumatized organ of Corti and lateral wall. ***c***, ***d***, Noise exposure for 1 h at the level of 106 dB SPL triggers the upregulation of c-Jun expression and S73 phosphorylation in the organ of Corti and lateral wall. ***c'***, ***d'***, In the lateral wall, c-Jun activation is most prominent in the root cell area. ***e***, Four hours of noise at 106 dB SPL shifts S73 phosphorylation in the lateral wall to stria vascularis and underlying fibrocytes. ***e'***, Closer examination shows that c-Jun is phosphorylated mainly in basal cells, in addition to fibrocytes. Strial cell layers are depicted and color coded above the histological image: Basal cells (blue); intermediate cells (yellow) together with endothelial cells lining capillaries (red); border cells (green). ***e''***, In the basalmost, most damaged part of the cochlea, border cells and intermediate cells of stria vascularis also show S73 phosphorylation. ***f–g'***, Following the acute induction, the c-Jun stress response largely disappears from the organ of Corti and lateral wall between 6 and 22 h. ***h***, Schematic presentation of the suggested potassium-recycling route in the cochlea. Color codes: outer hair cells (blue); Deiters’, Hensen, Claudius, and root cells (green); fibrocytes (yellow); stria vascularis (red). ***i***, ***j***, Noise exposure fails to upregulate c-Jun expression and its phosphorylation in nonsensory cells of *Gfi1*-depleted cochleas that lack most hair cells. All panels except ***e''*** are from the 45–50 kHz region of the cochlea. Fi, Fibrocytes; OC, organ of Corti; RC, root cell; SM, scala media; SV, stria vascularis. Scale bar: (in ***j***) ***a–g***, ***i***, ***j***, 20 µm; (in ***j***) ***c'***, ***d'***, ***e'***, ***e''***, ***f'***, ***g'***, 45 µm.

**Figure 4.**
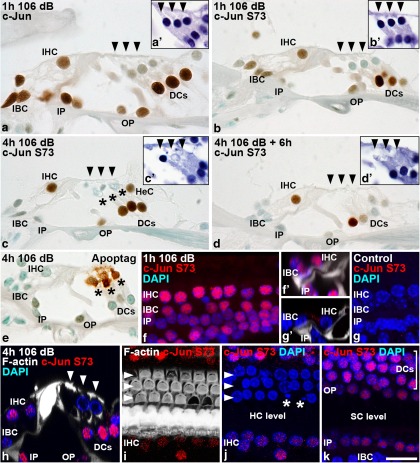
Acoustic overstimulation triggers the c-Jun stress response in the organ of Corti. ***a–d***, Dynamics of c-Jun expression and S73 phosphorylation in the organ of Corti after noise exposure, which are shown in paraffin sections prepared at various postexposure time points. Arrowheads mark outer hair cells. The c-Jun stress response is restricted to nonsensory cells and inner hair cells, while it is absent in vulnerable outer hair cells. Note the seemingly intact, degenerating or lost status of outer hair cells at various time points. Dying outer hair cells are visible (***c***, asterisks). Note also that c-Jun phosphorylation is particularly transient in inner border cells around inner hair cells, as these cells are not anymore phosphorylated at the end of the 4-h-long noise exposure. See Results for details. ***a'***, ***b'***, ***c'***, ***d'***, Degeneration status of outer hair cells is shown in hematoxylin-stained sections that are consecutive to the sections prepared for immunohistochemistry. ***e***, Outer hair cells show prominent ApopTag staining immediately after noise exposure. ***f***, Consistent with paraffin sections, whole-mount specimens imaged under confocal microscopy show c-Jun S73 induction in inner hair cells, inner border cells, and inner pillar cells acutely after a 1-h-long noise exposure. ***f'***, Side view of the corresponding confocal stack. DAPI stains cell nuclei. Phalloidin labels the F-actin cytoskeleton of pillar cells. ***g***, ***g****'*, In corresponding views from nonexposed specimens, c-Jun S73 expression is absent. ***h***, Consistent with paraffin sections, immediately after a 4-h-long exposure, inner hair cells as well as supporting cell populations, except for inner border cells, are positive for the c-Jun S73 signal, shown in a side view. ***i***, ***j***, Immediately after a 4-h-long exposure, a surface view from the level of hair cells shows that neither surviving outer hair cells nor these cells with apoptotic profiles (asterisks) express phosphorylated c-Jun. Note that nuclear fragmentation is located in the area where the F-actin-rich apical domain of the corresponding outer hair cell is missing. Inner hair cells do show c-Jun S73 staining. Arrowheads mark the three outer hair cell rows. ***k***, Surface view from the supporting cell level of the same specimen shows c-Jun phosphorylation in Deiters’ and pillar cells, but not anymore in inner border cells. DC, Deiters’ cell; HeC, Hensen’s cell; HC, hair cell; IBC, inner border cell; IP, inner pillar cell; OP, outer pillar cell; SC, supporting cell. Scale bar: (in ***k***) ***a–k***, 10 µm.

Knowledge about the c-Jun N-terminal phosphorylation pattern in the acoustically injured cochlea might provide insights into the mechanisms of stress-induced JNK/c-Jun signaling. Thus, CBA/Ca mice were exposed for 1 or 4 h to 8-16 kHz octave-band noise at 106 dB SPL. We focused histological analysis on the 45–50 kHz region, located in the upper basal turn of the cochlea (Figs. 3, 4). This region is more sensitive to environmental stressors than apical turns. Immediately after both 1-h-long and 4-h-long noise exposures, the 45–50 kHz region showed partial OHC loss, and several of the existing OHCs had morphological signs of degeneration (Fig. 4*a'*,*b'*,*c'*). IHCs appeared to be histologically intact. Light microscopic analysis did not show obvious morphological changes in hair cells located in the upper levels of the cochlea (data not shown).

Immediately after a 1 h 106 dB SPL exposure, c-Jun expression and c-Jun S73 phosphorylation were induced in the organ of Corti and in the root cell area of the lateral wall (Fig. 3*c–d'*). After 4 h of continuous noise, c-Jun S73 had disappeared from the root cell area, but had proceeded to upper areas of the lateral wall, namely to fibrocytes and the innermost cell layer of stria vascularis (Fig. 3*e*,*e'*). This phosphorylation in the lateral wall disappeared by 22 h postexposure (Fig. 3*f–g'*). In the 45–50 kHz region, the outermost cell layer of stria vascularis, which is responsible for potassium secretion into the endolymph, was only weakly positive for c-Jun S73. In contrast, this strial cell layer showed strong c-Jun S73 phosphorylation in the basalmost, most strongly affected region of the cochlea (Fig. 3, compare *e'*, *e''*). Thus, upon noise exposure, c-Jun is acutely upregulated and phosphorylated in the potassium-recycling route that extends from the nonsensory cells of the organ of Corti to the lateral wall (Fig. 3*h*). This temporal sequence of c-Jun activation correlates with the known sequence of potassium ion transport along this recycling route (Kikuchi et al., 2000), suggesting that these processes might be interconnected.

To investigate whether functional hair cells, which are the principal entry site of potassium into the organ of Corti, are needed for c-Jun activation in supporting cells and in the cells of the lateral wall, adult *Gfi1*-depleted mice, which lack most of the hair cells, were analyzed. After exposure for 1 h to 106 dB SPL, cochleas were immediately prepared for analysis. Upregulation of c-Jun expression and phosphorylation were absent from the organ of Corti and lateral wall of these mutant animals, showing that stress-induced c-Jun activation depends on the presence of OHCs (Fig. 3*i*,*j*).

We next compared the dynamics of c-Jun S73 phosphorylation and OHC loss in the noise-exposed organ of Corti. Deiters’ cells, of which a large part showed constitutive c-Jun expression in unexposed cochleas (Fig. 3*a*), showed acute and robust c-Jun S73 induction in response to a 106 dB exposure for 1 h. This phosphorylation occurred concomitantly with the upregulation of basal c-Jun expression (Fig. 4*a*,*b*). c-Jun S73 induction was also seen in pillar cells, in a part of the IHCs, and in the supporting cells that surround IHCs (termed here collectively as inner border cells; Fig. 4*a*,*b*). After 4 h of continuous noise and analysis immediately thereafter, c-Jun S73 phosphorylation was maintained in Deiters’ cells, pillar cells, and IHCs, but was downregulated in inner border cells (Fig. 4*c*). Six hours after this noise exposure, c-Jun phosphorylation in Deiters’ cells, pillar cells, and IHCs had further decreased (Fig. 4*d*), and at 22 h postexposure, it was absent (Fig. 3*g*). Notably, while a part of the IHCs showed acute c-Jun upregulation and phosphorylation, degenerating OHCs were negative for c-Jun and c-Jun S73 at each time point studied (Fig. 4*a–c*). ApopTag staining on paraffin sections revealed dying OHCs, whereas IHCs did not show this staining and lacked morphological signs of degeneration (Fig. 4*e*). This positive staining in OHCs suggests that their death has features of apoptosis. The results on c-Jun S73 immunoreactivity obtained in paraffin sections were confirmed in whole-mount specimens. Whole mounts showed stress-induced, acute c-Jun S73 phosphorylation in IHCs, inner border cells (Fig. 4*f–g*), pillar cells, and Deiters’ cells, but not in OHCs (Fig. 4*h–k*). Together, these results suggest that c-Jun phosphorylation is not part of an intrinsic apoptotic process in adult OHCs and, on the other hand, that c-Jun phosphorylation in IHCs does not direct these cells to death.

As c-Jun phosphorylation was not detected in OHCs after 1- and 4-h-long noise exposures, we studied whether these cells phosphorylate c-Jun anyway in a very abrupt fashion. Mice were exposed for 15 min to 106 dB SPL, and cochleas were prepared for analysis immediately thereafter. OHCs did not show c-Jun S73 or c-Jun S63 immunostaining after this short exposure, arguing against momentary c-Jun activation in response to noise stress (data not shown).

In addition to histological analysis from the 45–50 kHz region (Figs. 3, 4), cytocochleograms were generated to reveal the longitudinal pattern of OHC loss and c-Jun phosphorylation in the organ of Corti of the cochlea (see Fig. 7*a–d*). Cytocochleograms demonstrate OHC loss in the basal part of the cochlea and that c-Jun S73 phosphorylation is concentrated in this area, yet to cells other than OHCs. Cytocochleograms reveal not only rapid induction, but also rapid downregulation of this phosphorylation after noise exposure.

### Ototoxic stress triggers c-Jun phosphorylation in the organ of Corti and lateral wall

**Figure 5.**
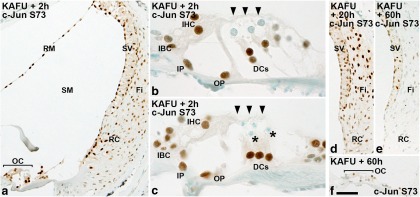
Ototoxic trauma triggers a robust c-Jun stress response in the cochlea. ***a***, Two hours after ototoxin injections, widespread c-Jun S73 immunoreactivity is seen throughout the lining of scala media, in all three layers of stria vascularis, and in fibrocytes and root cells of the lateral wall, in addition to the organ of Corti. ***b***, ***c***, In the organ of Corti, supporting cells and inner hair cells show c-Jun S73 immunoreactivity. Degenerating outer hair cells (***b***, arrowheads) and their apoptotic remnants (***c***, asterisks) lack this phosphorylation. ***d***, ***e***, ***f***, In the lateral wall (***d***, ***e***) and in the organ of Corti (***f***), the c-Jun stress response is downregulated to low levels by 60 h postexposure. DC, Deiters’ cell; Fi, fibrocyte; IBC, inner border cell; IP, inner pillar cell; KAFU, kanamycin and furosemide; OC, organ of Corti; OP, outer pillar cell; RC, root cells; RM, Reissner’s membrane; SM, scala media; SV, stria vascularis. Scale bar: (in ***f***) ***a***, ***d–f***, 36 µm; ***b***, ***c***, 10 µm.

To study the impact of ototoxic drugs on c-Jun activation, mice were exposed to the aminoglycoside antibiotic kanamycin and the loop-diuretic furosemide, whose synergistic effect triggers rapid and widespread OHC loss (Oesterle et al., 2008; Taylor et al., 2008; Anttonen et al., 2012, 2014). This effect is based on the direct damaging effect of aminoglycosides on hair cells and on the disruptive effect of loop-diuretics on potassium secretion from stria vascularis into endolymph. Two hours after drug injections, extensive OHC loss was already found at the 45–50 kHz level of the cochlear duct. Correspondingly, the organ of Corti and lateral wall at this level displayed robust c-Jun upregulation and phosphorylation (Fig. 5*a*). In the organ of Corti, c-Jun activation was seen in IHCs and supporting cells prior to, during, and after OHC degeneration. OHCs themselves were negative for c-Jun and c-Jun S73 at each stage (Fig. 5*b*,*c*). Ototoxins triggered widespread c-Jun activation in stria vascularis and fibrocytes of the lateral wall, and in other regions of the epithelial lining of the endolymphatic space (Fig. 5*a*). These observations link c-Jun phosphorylation to impaired ion homeostasis in the ototoxically lesioned cochlea. Similarly, as in the case of noise exposure, OHCs showed ApopTag labeling following kanamycin-furosemide exposure, suggesting that the death of these cells has features of apoptosis (data not shown).

Cytocochleograms prepared from ototoxin-challenged cochleas (see Fig. 9) show that c-Jun phosphorylation was not restricted to the vulnerable basal portion of the cochlea, but was seen in its upper portion as well, reflecting the severity of the insult. Based on histological views and cytocochleograms (Fig. 5*d–f*; see Fig. 9), broad c-Jun S73 immunoreactivity was still maintained at 20 h postexposure, but was for the most part abolished by 60 h postexposure. These dynamics of c-Jun phosphorylation follow the time course reported for aminoglycoside accumulation into the different compartments of the cochlea after systemic drug application (Imamura and Adams, 2003). Together, both trauma models applied trigger an acute c-Jun stress response in the nonsensory cells and IHCs of the organ of Corti, and in the cells of the lateral wall. Interestingly, these cells of the organ of Corti are not killed by the traumas, as opposed to OHCs lacking the c-Jun stress response.

### c-Jun phosphorylation is a biomarker for harmful noise levels

**Figure 6.**
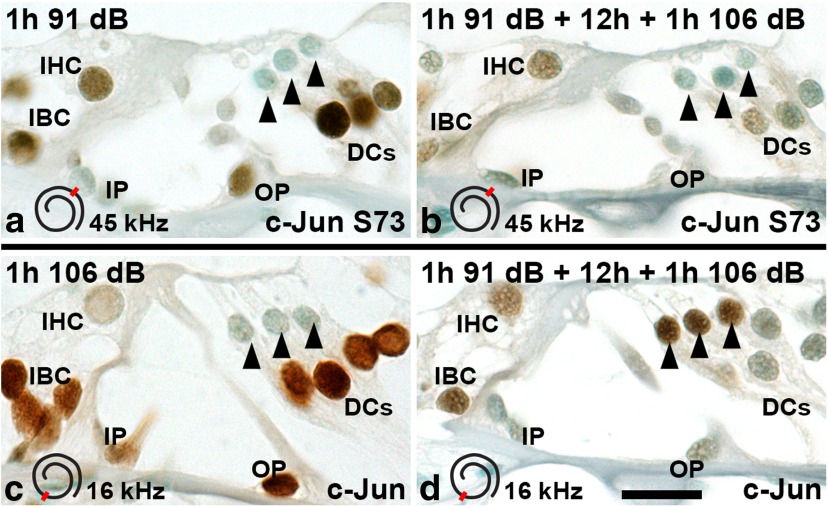
Sound preconditioning attenuates the c-Jun stress response. Immunohistochemical detection of c-Jun expression and S73 phosphorylation in the organ of Corti after various acoustic overexposure paradigms. The spatial location along the cochlear duct of the area displayed is shown in an inset in each image. ***a***, Exposure for 1 h at 91 dB SPL induces c-Jun phosphorylation comparable to exposure to 106 dB (compare with Fig. 4*b*). ***b***, Sound preconditioning (see Results for details) attenuates c-Jun phosphorylation, most clearly in Deiters’ cells located around the vulnerable outer hair cells. ***c***, In upper levels of the cochlear duct, exposure for 1 h at 106 dB upregulates c-Jun in supporting cells, in a manner similar to that in lower turns of the cochlea (compare with Fig. 4*a*). ***d***, In upper levels of the cochlear duct, sound preconditioning limits c-Jun upregulation in supporting cells, in a manner similar to that in lower levels (***b***), but triggers c-Jun expression in intact-appearing outer hair cells (arrowheads). DC, Deiters’ cell; IBC, inner border cell; IP, inner pillar cell; OP, outer pillar cell. Scale bar: (in ***d***) ***a–d***, 10 µm.

**Figure 7.**
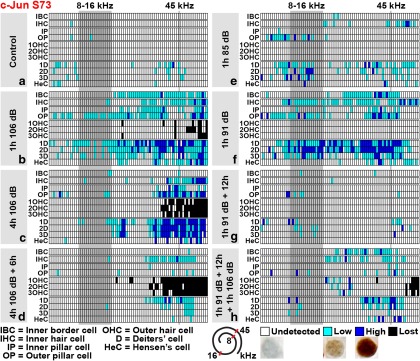
Cytocochleograms displaying c-Jun S73 phosphorylation in the organ of Corti along the cochlea after various noise paradigms. ***a–h***, For each noise paradigm, a cytocochleogram generated from one cochlea that represents the average expression pattern is shown. The tonotopic organization of the organ of Corti is displayed from left to right (from apical to basal turns). The noise band used (8–16 kHz) is shown as a gray column, similar to the approximate region (45 kHz) from where most histological views of the current study have been obtained. These sites are also marked in the schematic presentation of the spiraling organ of Corti. Abbreviations and color codes are listed below the grids. Representative examples of weak and strong c-Jun S73 immunostaining are shown. See Materials and Methods for details.

If c-Jun N-terminal phosphorylation reflects a cellular stress response, the magnitude of the response might differ between damaging and nondamaging insults. To test this, mice were exposed to noise for 1 h to 85 or 91 dB SPL, and cochleas were analyzed immediately thereafter. In addition to histological sections (Fig. 6*a*), cytocochleograms along the length of the cochlear duct were used for analysis (Fig. 7*e–g*). Prior data have shown that 85 and 91 dB SPL exposures trigger neither hair cell loss nor the loss of ribbon synapses between IHCs and afferent nerve fibers, although they cause temporary hearing threshold shifts (Housley et al., 2013; Fernandez et al., 2015). Consistent with these data, we did not find OHC loss after these exposures (Fig. 7*e–g*, cytocochleograms). The 91 dB SPL was strong enough to trigger c-Jun expression and phosphorylation in the organ of Corti and lateral wall (Fig. 6*a*; data not shown). Both immunoreactivities after 91 dB exposure had largely comparable pattern and intensity as seen after 106 dB SPL exposure for 1 h (Fig. 7*b–f*, compare cytocochleograms; compare Figs. 6*a*, 4*b*, histological views). In contrast, as demonstrated in the cytocochleograms (Fig. 7), c-Jun phosphorylation was minimal after the 85 dB SPL exposure. Following this low-level noise, weakly c-Jun S73-positive cells were located in a scattered manner rather than being accumulated to a damage area, as was seen upon louder noise exposures (Fig. 7*b*,*e*, compare cytocochleograms). Thus, in addition to SPLs causing permanent structural damage, c-Jun is phosphorylated upon noise levels that leave the cochlea light microscopically intact, but cause a transient decrease in hearing sensitivity. Therefore, c-Jun phosphorylation is a biomarker for cellular stress-evoking noise levels.

### Acoustic preconditioning modulates stress-induced c-Jun phosphorylation in the organ of Corti

Preconditioning with low-to-moderate noise levels protects hearing against a subsequent, more intense insult, provided that the interval between the exposures is less than ∼24 h (Yoshida and Liberman, 2000). The mechanism behind this “toughening” event is unresolved. As our results revealed that c-Jun S73 phosphorylation is not only an acute, but also a transient, response to noise stimulation, we hypothesized that the mechanisms directing rapid c-Jun silencing might still be active when a second insult is applied. To study this, mice were preconditioned for 1 h at 91 dB SPL. After 12 h, they were exposed for 1 or 4 h to 106 dB SPL, and cochleas were immediately thereafter prepared for analysis. Preconditioning resulted in a clear attenuation of the extent and intensity of c-Jun S73 phosphorylation, particularly in Deiters’ cells of the organ of Corti, shown in histological views from the 45–50 kHz region (compare Figs. 6*b*, 4*b*) and in cytocochleograms (Fig. 7, compare *b*, *f–h*). Thus, sound preconditioning limits stress-induced c-Jun phosphorylation in supporting cells that surround the death-prone OHCs, suggesting that dampening of the c-Jun stress response is part of the protective mechanism of acoustic preconditioning.

**Figure 8.**
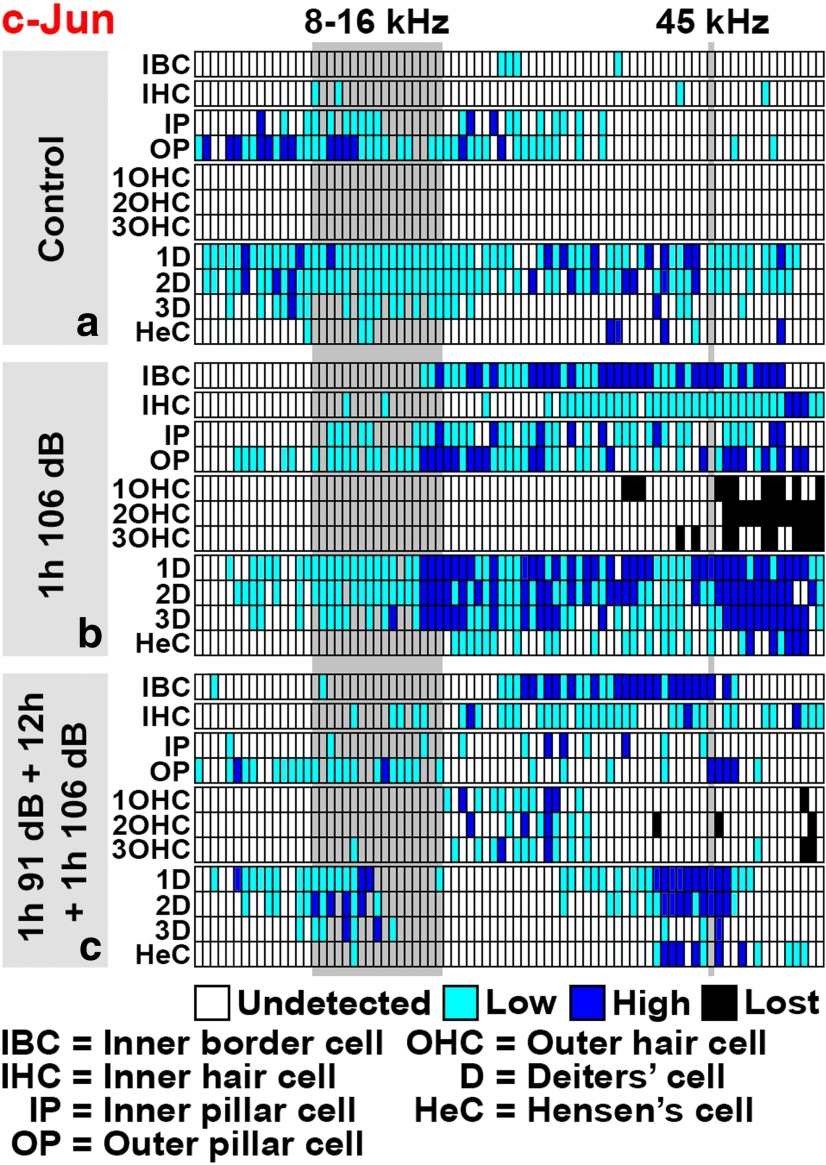
Cytocochleograms displaying c-Jun expression in the organ of Corti along the cochlea after various noise paradigms. ***a–c***, See Figure legend 7 and Materials and Methods for details. Note that acoustic preconditioning leads to upregulation of c-Jun expression in outer hair cells, but exclusively at the level above the trauma area (***c***).

As described above, c-Jun expression was absent from OHCs challenged with a single 1 h 106 dB SPL exposure (Figs. 4*a*, 8*b*). Unexpectedly, c-Jun expression was upregulated in a part of OHCs of acoustically preconditioned cochleas. This c-Jun upregulation was found after preconditioning for 1 h at 91 dB SPL, followed by a recovery of 12 h, and then exposure for 1 h at 106 dB SPL (Fig. 6*c*,*d*) or 4 h (data not shown). Analysis was performed immediately after noise exposures. Notably, along the length of the cochlear duct, c-Jun was upregulated in the 16–45 kHz region (i.e. above the level where OHCs were lost), as shown in cytocochleograms (Fig. 8*b*,*c*). In this region, approximately 25% of OHCs expressed c-Jun. Only a minor fraction of these cells showed c-Jun S73 phosphorylation (Fig. 7*h*). Notably, this 16–45 kHz region did not show OHC loss, and the c-Jun-expressing OHCs lacked morphological signs of degeneration (Fig. 6*d*). Therefore, an apoptotic fate for these cells cannot be suggested. We conclude that c-Jun expression can be upregulated in OHCs upon recurrent acoustic stress. It might be connected with stress-induced metabolic alterations in these cells. As this c-Jun upregulation in OHCs was only minimally associated with N-terminal phosphorylation, its function might be JNK-independent. Whether this intriguing event after preconditioning is beneficial or harmful for OHC function remains unanswered.

**Figure 9.**
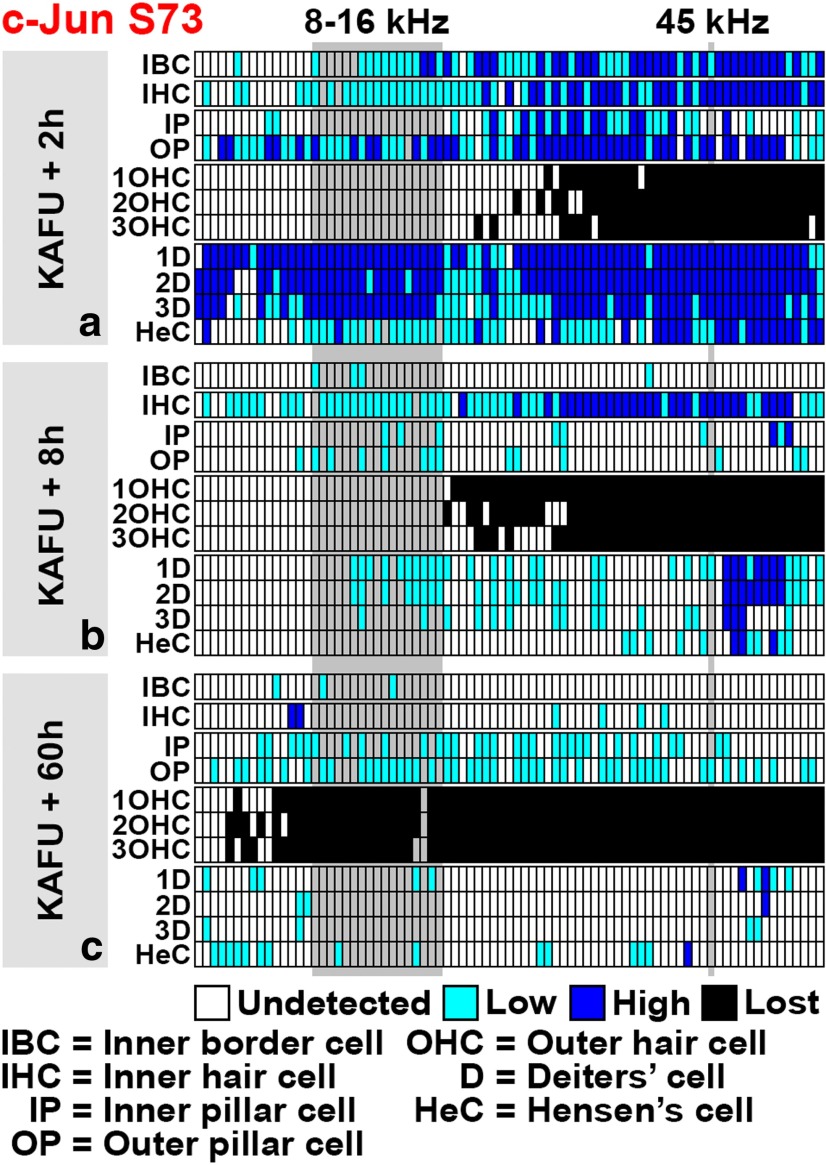
***a–c***, Cytocochleograms displaying c-Jun S73 phosphorylation in the organ of Corti along the cochlea after an ototoxic insult. See Figure legend 7 and Materials and Methods for details. ***a***, Two hours after kanamycin and furosemide injections, c-Jun is strongly phosphorylated in supporting cells and inner hair cells. This response is more widespread than the one induced by acoustic trauma (compare with Fig. 7*b*). ***b***, Intensity of c-Jun S73 immunostaining in supporting cells decreases rapidly, despite the progression of outer hair cell loss toward the apical turn. Inner hair cells still show strong c-Jun S73 signal at 8 h postexposure. ***c***, At 60 h postexposure, outer hair cell loss has reached the apical turn. At this time point, c-Jun S73 is found only in scattered cells in the organ of Corti.

### Genetic blockade of c-Jun phosphorylation partially protects outer hair cells from acoustic trauma

To find out whether the blockade of c-Jun N-terminal phosphorylation confers OHC protection from an acoustic insult, we studied the *JunAA/AA* knock-in mice in which the N-terminal serines 63 and 73 have been replaced by alanines (Behrens et al., 1999). Thus, in these animals, stress-induced JNKs cannot trigger c-Jun N-terminal phosphorylation

**Figure 10.**
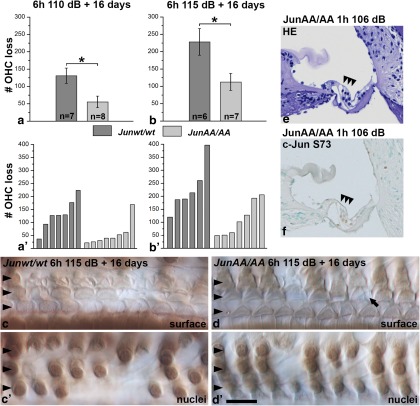
*JunAA/AA* mice are partially protected from noise-induced outer hair cell (OHC) loss. ***a***, ***b***, Histograms show the mean ± SEM of outer hair cell loss in *Junwt/wt* (dark gray) and *JunAA/AA* (light gray) mice 16 days after a 6-h-long noise exposure at 110 or 115 dB SPL. At both noise levels, *JunAA/AA* mice show a statistically significant attenuation of OHC loss (110 dB, *p = 0.01705; 115 dB, *p = 0.02527). The numbers of cochleas used for this analysis are shown. ***a'***, ***b'***, OHC loss is shown for each individual cochlea used for the histograms in ***a*** and ***b***. ***c–d'***, Noise-exposed cochleas from *Junwt/wt* (***c***, ***c'***) and *JunAA/AA* (***d***, ***d'***) mice embedded in resin and viewed under DIC optics at the 45 kHz region. Arrowheads point OHCs. The same hair cells/sites of lost hair cells are viewed at the surface and nuclear levels. In these views, 3 and 12 OHCs, respectively, are lost in *JunAA/AA* and *Junwt/wt* cochleas. Note that epithelial wound healing (scar formation) is comparable in these genotypes. Arrow shows an example of normal scar formation in *JunAA/AA* mice. ***e***, Paraffin-embedded and hematoxylin-stained cross section from the 45 kHz region of a noise-exposed *JunAA/AA* cochlea shows unaltered overall morphology. Arrowheads point to OHCs. ***f***, Noise-exposed *JunAA/AA* cochleas show insignificant c-Jun S73 immunostaining, indicating the specificity of the antibody used. Very weak staining is seen in Deiters’ and pillar cells, where c-Jun is constitutively expressed and is strongly upregulated upon traumas. Scale bar: (in ***d'***) ***a–d'***, 10 µm; ***e***, ***f***, 65 µm.

**Figure 11.**
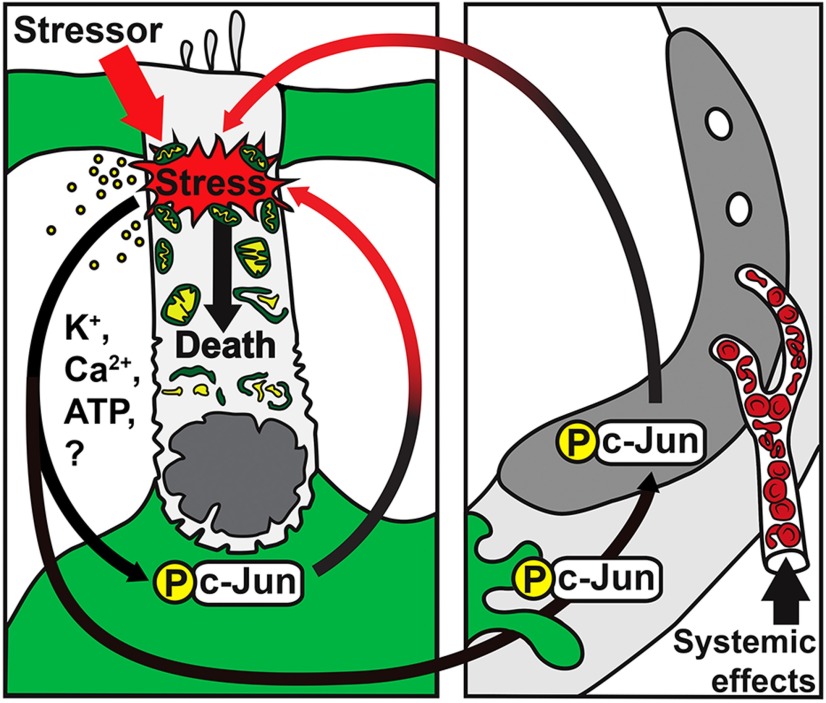
Hypothetical model of the c-Jun stress response in the traumatized, adult cochlea. The model is based on the pattern of c-Jun N-terminal phosphorylation upon noise and ototoxic exposures. These stressors induce direct stress on outer hair cells (OHCs). Supporting cells (green) are able to perceive this stress, perhaps through factors released by stressed OHCs (Housley and Gale, 2010), leading to c-Jun phosphorylation in supporting cells. c-Jun is sequentially phosphorylated in the lateral wall (right picture). This temporal sequence follows the suggested sequence of potassium ion recycling within the nonsensory cell compartments and fluids of the cochlea. c-Jun phosphorylation in nonsensory cells might induce additional stress (black-to-red arrows) on OHCs through unknown paracrine mechanisms. Once intolerable levels of cellular stress are reached, OHC death is stimulated. By inhibiting c-Jun phosphorylation (*JunAA/AA* mice), formation of this additional stress on OHCs can be decreased, leading to increased OHC survival. Besides local cochlear events, systemic factors, such as stress hormones, may contribute to the induction of the c-Jun stress response in the cochlea.

*JunAA/AA* and *Junwt/wt* mice were exposed to noise for 6 h at 110 or 115 dB SPL to achieve an OHC loss large enough for quantification purposes. This genetic intervention led to partial, but significant, OHC protection, as evidenced 16 days after exposure by counting OHC numbers in resin-embedded whole-mount specimens (Fig. 10*a–d'*). These specimens also showed that the *JunAA/AA* mutation does not affect the scar formation ability of supporting cells at the epithelial surface (Fig. 10*c–d'*). Neither whole-mount specimens nor paraffin sections (Fig. 10*e*) revealed loss of supporting cells, indicating that trauma-induced c-Jun N-terminal phosphorylation does not promote their survival. In contrast to noise-exposed control cochleas, the induction of c-Jun S73 immunostaining was insignificant in noise-exposed *JunAA/AA* cochleas, demonstrating the specificity of the antibody used (Fig. 10*f*). Together, these results suggest that JNK/c-Jun activation mediates at least part of the detrimental effects of loud noise that causes OHC loss. A hypothetical model of how c-Jun phosphorylation mediates OHC loss acutely after noise and ototoxic drug exposures is presented in Fig. 11.

## Discussion

Activation of the JNK/c-Jun pathway connects cellular stress with various physiological outcomes. Apoptotic outcome has been emphasized in a wide range of pathological conditions, particularly in neurons and other sensory cells. Nevertheless, the JNK/c-Jun stress response is not simply linked to cell death, as shown, for example, in the injured brain, where only a fraction of neurons with this stress response die (Yang et al., 1997; Herdegen et al., 1998; Brecht et al., 2005). In glial cells, JNK/c-Jun activation triggers neurotoxic inflammation (Hidding et al., 2002; Lund et al., 2005; Waetzig et al., 2005; Eggert et al., 2010). In the cochlea, JNK/c-Jun signaling has been linked to hair cell-intrinsic death following traumas (Pirvola et al., 2000; Ylikoski et al., 2002; Wang et al., 2003). Contrary to this somewhat restricted view, the present study suggests that the mode of action of JNK/c-Jun signaling in the traumatized cochlea is not as straightforward as previously believed. Furthermore, although pharmacological inhibition of this signaling has been shown to confer protection against hair cell and hearing loss, direct genetic evidence is lacking. We have addressed these issues in the current study and used N-terminally phosphorylated c-Jun as an indicator of stress-induced nuclear JNK/c-Jun activation. We refer to this as the c-Jun stress response in the discussion below.

### The c-Jun stress response during cochlear development

c-Jun N-terminal phosphorylation was induced in the GER of the developing cochlea at the stage when this structure is disappearing by apoptosis, highlighting the involvement of JNK/c-Jun activation in “classic” programmed cell death (Sun et al., 2005). During development, c-Jun phosphorylation was also induced in *Gfi1*-depleted, degenerating cochlear hair cells, demonstrating that the c-Jun stress response can be induced cell-intrinsically in differentiating hair cells by the withdrawal of a survival factor. This is also consistent with the known role of JNK/c-Jun signaling in immature neurons deprived of trophic factors (Watson et al., 1998).

### c-Jun N-terminal phosphorylation is a biomarker for cellular stress and altered ion homeostasis in the adult cochlea

Upon exposure to environmental stressors, tissue homeostasis is in danger, and the cellular stress response is activated. If stressful stimulus does not exceed a certain threshold, cells can cope with it by mounting appropriate protective processes. If the stressful agent is too strong, activated stress signaling is integrated into cell death pathways. Our results show that c-Jun N-terminal phosphorylation is an excellent biomarker for cellular stress in the traumatized adult cochlea, irrespective of the final cellular fate. First, upon noise exposure, c-Jun phosphorylation extended from the 8–16 kHz noise band down to the base of the cochlea, but the strongest and most homogenous phosphorylation was localized to the region of morphological damage. Second, based on experiments with various noise intensities, c-Jun also became phosphorylated at lower noise levels that leave the cochlea light microscopically intact. This is what one would expect from a stress response factor, as these “nondamaging” noise levels are also known to cause temporary impairment in hearing function and fine-grained structural defects in hair cell stereociliary bundles, which were not examined in our study (Liberman and Dodds, 1987; Wang et al., 2002). Third, connected to the fact that acoustic overstimulation modulates potassium trafficking from the organ of Corti to the lateral wall (Wangemann, 2006), c-Jun was dynamically phosphorylated after noise exposure along this ion-trafficking route. This suggestion that c-Jun N-terminal phosphorylation indicates imbalances in ion homeostasis was even more clearly evidenced in ototoxin-exposed cochleas. Furosemide, one of the ototoxic compounds used, blocks potassium secretion into the endolymph. This was reflected as a c-Jun stress response throughout the epithelial lining of the endolymphatic space.

### The c-Jun stress response in death-resistant cells of the adult cochlea

Upon exposure to noise and ototoxic drugs, the adult organ of Corti displayed an unexpected pattern of c-Jun N-terminal phosphorylation. It was absent from the vulnerable OHCs, but was induced in the death-resistant IHCs and supporting cells. Thus, the function of the c-Jun stress response is something other than the regulation of cell-intrinsic apoptosis. In adult mice, c-Jun was phosphorylated in all supporting cell types, but most prominently in Deiters’ cells. This robust c-Jun induction appears to be linked with the constitutive c-Jun expression in these cells. Although OHCs lacked the c-Jun stress response, OHC presence was, nevertheless, required for c-Jun activation in adjacent supporting cells, as evidenced in the adult, *Gfi1*-depleted mice. They lacked cochlear hair cells and did not phosphorylate c-Jun in Deiters’ cells after noise exposure. This absence of the c-Jun stress response in the *Gfi1* mutant mice is consistent with the fact that hair cells are the primary sensors of acoustic signals and the entry site for potassium into the organ of Corti. As the *Gfi1* mutant mice lacked c-Jun phosphorylation in the lateral wall as well, activation of the stress response at this site may be a consequence of homeostatic imbalances originating from the organ of Corti.

What is the role of the c-Jun stress response in nonsensory cells of the cochlea? We cannot exclude the possibility that after mild traumas and in the more resistant upper portion of the cochlea, it has beneficial effects by striving for homeostatic balance. However, c-Jun phosphorylation was concentrated to the lower portion of the cochlea that showed morphological damage, suggesting that the stress response has harmful effects there, at least after a certain stimulus threshold has been exceeded. In this affected region, JNK/c-Jun activation in nonsensory cells might mediate OHC degeneration, perhaps in an integrative manner with hair cell-intrinsic mechanisms, such as oxidative stress. The findings of c-Jun phosphorylation in supporting cells prior to and during OHC degeneration support this suggestion. Accumulating data highlight the intimate crosstalk between hair cells and supporting cells (May et al., 2013; Monzack and Cunningham, 2013). The current results support these data and are consistent with the proposed role of JNK/c-Jun signaling in glial cell-neuron interactions in the CNS (Hidding et al., 2002; Lund et al., 2005; Waetzig et al., 2005; Eggert et al., 2010).

In addition to supporting cells, c-Jun was phosphorylated in a part of IHCs, both upon noise and ototoxic challenges. Similar to supporting cells, this c-Jun activation was not associated with a death-prone cellular fate. One possibility is that c-Jun phosphorylation in IHCs mediates stress on ribbon synapses that connect these cells to the afferent nerve fibers. This possibility is made relevant by taking into account that both the c-Jun stress response (current results) and ribbon synapse damage (Kujawa and Liberman, 2009; Liberman et al., 2015) are triggered rapidly following noise exposure. Further, the most transient c-Jun phosphorylation in the supporting cell population below IHCs, comprising the inner border cells, is of high interest. As these cells are responsible for glutamate uptake from the synaptic region, the c-Jun stress response might be involved in mediating trauma-induced excitotoxicity. Together, ribbon synapse damage, which already occurs at moderate noise levels, might be linked to c-Jun phosphorylation, which is a spatiotemporally comparable event.

### Mechanisms of the c-Jun stress response in the adult cochlea

We did not detect c-Jun upregulation or N-terminal phosphorylation in the vulnerable OHCs of injured adult cochleas. How then does pharmacological MLK or JNK blockade attenuate OHC loss? One possibility is the one suggested above that JNK/c-Jun activation in cochlear nonsensory cells mediates OHC death. Another possibility is that, in OHCs, cytoplasmic JNKs promote apoptosis in a c-Jun-independent manner by directly acting on mitochondrial proapoptotic factors (Dhanasekaran and Reddy, 2008). In OHC nuclei, JNKs could also use other nuclear substrates than c-Jun, although c-Jun is the best-known JNK substrate in promoting transcription-dependent apoptosis (Besirli et al., 2005; Björkblom et al., 2008). Due to the lack of reliable phospho-JNK antibodies that work in immunohistochemistry, we were unable to exclude a possible c-Jun-independent role of JNKs in OHCs. Our results suggest that the c-Jun stress response in nonsensory cells of the adult cochlea has a detrimental potential on OHCs. In support of this suggestion, *JunAA/AA* mice with mutated c-Jun N-terminal phosphoacceptor sites showed partial, but significant, attenuation of OHC loss following noise exposure. With this first genetic evidence of OHC protective potential of JNK/c-Jun inhibition, we provide a novel insight that the c-Jun stress response mediates, in part, the death of OHCs in a paracrine fashion.

### The c-Jun stress response as part of the acoustic preconditioning process

Several studies have shown that preconditioning by sound or restraint stress protects against hearing loss triggered by subsequent, more intense trauma. Decreased hair cell loss has also been shown after preconditioning (Roy et al., 2013). The underlying molecular mechanisms are, however, elusive. It has been suggested that preconditioning triggers protective responses, such as the upregulation of heat shock proteins (HSPs; Yoshida et al., 1999). Consistent with the fact that HSPs silence JNK/c-Jun signaling in several cellular models (Mosser et al., 1997), pharmacological HSP upregulation antagonizes JNK phosphorylation in aminoglycoside-treated cultures of vestibular organs of the inner ear (Francis et al., 2011). However, the vestibular cell type where JNK activation occurred was not shown in that study. The current study shows that sound preconditioning attenuates the c-Jun stress response in supporting cells of the cochlea. Thus, while our study does not reveal the possible interactions between JNK/c-Jun signaling and HSPs, our results, together with prior data (Roy et al., 2013), suggest that one mechanism of preconditioning-conferred hearing protection is the attenuation of hair cell death via suppression of the c-Jun stress response.

### Toward therapeutic auditory hair cell protection through inhibition of the c-Jun stress response

Understanding the mode of action of JNK/c-Jun signaling in the injured cochlea is important, taking into account the current aim to develop a pharmacological therapy against hearing loss by inhibiting the interaction between JNKs and their targets (Borsello et al., 2003; Suckfuell et al., 2014). JNKs and c-Jun have pleiotropic actions under both physiological and pathophysiological conditions; harmful in mediating stress-induced cell death and undesirable inflammation, and beneficial, for example, in promoting neuronal repair after peripheral axotomy (Raivich, 2008). As widespread JNK/c-Jun activation is known to occur in the stressed nervous system studies focusing on this activation in the whole stressed auditory pathway, together with functional measurements, is an important future step to be taken. The current study shows that the c-Jun stress response is activated acutely and transiently in the cochlea, suggesting that the time window for therapeutic actions is limited, consistent with the fact that most significant OHC loss occurs within 1 d after trauma. Our results also suggest that JNK/c-Jun activation is part of a generalized cellular stress response (Fig. 11) and that targeting its beneficial components, such as HSPs, could be one alternative way to modulate JNK/c-Jun activation and confer hair cell protection. Finally, the current study shows that the c-Jun stress response arises in cell types responsible for cochlear homeostasis and that genetic blockade of c-Jun N- terminal phosphorylation in these cells confers partial, but significant, protection of vulnerable OHCs.

## Synthesis

The decision was a result of the Reviewing Editor Darcy Kelley and the peer reviewers coming together and discussing their recommendations until a consensus was reached. A fact-based synthesis statement explaining their decision and outlining what is needed to prepare a revision is listed below. The following reviewer(s) agreed to reveal their identity: Lisa Cunningham

This manuscript provides new information about c-jun expression and activation (n-terminal phosphorylation) in the mouse cochlea under normal and stressed conditions. Overall the experimental data are convincing and they add to a growing body of knowledge suggesting that supporting cells play important roles in sensing and responding to hair cell stress and death. These results are interesting because small molecule inhibitors of the JNK-c-Jun pathway have been shown to protect against hair cell loss but the basic mechanism of JNK-c-Jun signalling in the cochlea has not been described before.

While it is unfortunate that the manuscript does not contain functional data on the hearing loss caused by the noise exposure and whether the sound preconditioning paradigm was indeed protective, the paper is sound and will be of interest to both scientists studying the inner ear and those studying glial responses to stress. The manuscript and figure preparation are excellent, especially the careful criteria described for "missing" hair cells and the development of a model in Figure 11.

There are two significant weaknesses

1. Mentioned above: the paper lacks data on the cochlear function in the mice exposed to noise, sound conditioning, and ototoxic drugs. The manuscript would be significantly stronger if it contained at least ABR measurements showing cochlear function in these mice. This is particularly true for the sound conditioning experiments, which are notorious for the biological variability in the protective response. Relevant to this, it appears that the sound preconditioning experiments done in this paper were performed in a genetic background strain in which sound preconditioning has not previously been shown to have a protective effect. While it is impossible to do those functional measures now (the animals have been euthanized), the authors should acknowledge and discuss this weakness and indicate how it affects the interpretation of the data.

2. The paper equates c-jun phosphorylation with JNK activation, which has been shown to contribute to hair cell death (much data from the Pirvola lab and others). JNK is not the only kinase that can activate c-jun (for example ERK can also phosphorylate c-jun), so c-jun phosphorylation cannot be taken as a direct readout of JNK activation. The paper needs to be clearer about this. For example, lines 404-405 and others.

Minor points:

1. Kanamycin + furosemide is a good model for hair cell death, but it is not a model for aminoglycoside-induced damage or hearing loss. It creates a much more severe lesion than aminoglycoside administration, and it does so with a much different time course. The manuscript should be clear about his.

2. The manuscript should indicate the ages of mice used for each experiment and the number of animals (not just number of cochleas) examined for each experiment. 7-8 weeks of age is a bit young for a sound preconditioning experiment, for example.

3. Line 199: "round window" should be "oval window"

4. Line 205: P12 is rarely considered a "mature cochlea"

5. Lines 237 and elsewhere: The "lower medial" turn of the mouse cochlea isn't the tonotopic location where 50 kHz is detected (according to the Eaton Peabody labs). Another reason to think the cochlear region examined was lower in frequency than 50 kHz is that it was the region of most severe damage after exposure to 8-16 kHz noise. I would expect the lesion from that noise exposure to be in the 8-24 kHz region. It would be helpful if the authors would clarify exactly where the analyses were performed along the cochlea and how they determined the frequency region they were examining.

6. My read of the literature indicates that the potassium recycling models in the cochlea are still controversial, particularly with regards to gap junctions (Jagger and Forge 2015; Wangemann 2006). Therefore the authors should use much more caution when linking the requirement for hair cells to potassium recycling or gap junctional coupling (examples lines 258, 310-311, 437-439).

7. Line 277 typo "reveled"

8. The paper jumps around in terms of presenting the results in the figures. For example, it discusses Figure 4 before Figure 3, and it jumps from describing Figure 4 to describing Figure 7. Similarly, it jumps from describing Figure 5 to describing Figure 9. This is confusing to the reader, and it would be helpful if the figures were discussed in the order in which they are presented.

9. Is it possible that c-jun phosphorylation is protective in the cells in which it is activated? As the Authors point out (lines 323-325), those cells don't die.

10. Lines 386-387: Why was it necessary to expose the mice to 110-115 dB noise? This should be clarified beyond the information provided.

9. Page 8: the authors discuss the specificity of the c-Jun phospho serine 73 antibody. Is the c-Jun phospho serine 73 immunostaining of the cochlea abolished in the JunAA/AA mice, in which serines 63 and 73 in c-Jun have been mutated to alanine ? If that is the case, it would demonstrate the specificity of the antibody.
